# Impact of Operating Table Height on the Difficulty of Mask Ventilation and Laryngoscopic View

**DOI:** 10.3390/jcm13195994

**Published:** 2024-10-08

**Authors:** Tsuyoshi Ikeda, Hirotsugu Miyoshi, Guo-Qiang Xia, Kenshiro Kido, Ayako Sumii, Tomoyuki Watanabe, Satoshi Kamiya, Soshi Narasaki, Takahiro Kato, Yasuo M. Tsutsumi

**Affiliations:** 1 Department of Anesthesiology and Critical Care, Hiroshima University Hospital, Hiroshima 734-8551, Japan; miyoshi0728@hotmail.co.jp (H.M.); kidoken46@gmail.com (K.K.); batakobatake@gmail.com (A.S.); ijaran7@gmail.com (S.N.); ta-katou@tf6.so-net.ne.jp (T.K.); yasuo223@hiroshima-u.ac.jp (Y.M.T.); 2 Department of Anesthesiology and Critical Care, Graduate School of Biomedical and Health Sciences, Hiroshima University, Hiroshima 734-8551, Japan; d243940@hiroshima-u.ac.jp; 3 Department of Anesthesiology, National Hospital Organization Kure Medical Center and Chugoku Cancer Center, Hiroshima 737-0023, Japan; nabe09075408227@yahoo.co.jp

**Keywords:** airway management, mask ventilation, laryngoscopic view, operating table height

## Abstract

**Background/Objectives**: Airway management techniques, including mask ventilation and tracheal intubation, are vital across medical settings. However, these procedures can be challenging, especially when environmental conditions are less than ideal. This study explores how the height of the operating table affects the difficulty of anesthesia techniques involving mask ventilation and tracheal intubation. **Methods**: Twenty anesthesiologists participated in this study. We assessed the difficulty of procedures such as mask ventilation, Macintosh laryngoscopy, and video laryngoscopy using McGRATH and AWS, on a four-level scale. The operating table’s height was adjusted at four points: the operator’s umbilicus, the inferior margin of the 12th rib, the xiphoid process, and the nipple. **Results**: Mask ventilation was easiest at the operating table’s height aligned with the inferior margin of the 12th rib. Conversely, direct laryngoscopic exposure was perceived as easier at higher table heights, with nipple height being optimal. The McGRATH laryngoscopy showed consistent difficulty across table heights, whereas the AWS tended to be somewhat more difficult at greater heights. **Conclusions**: The optimal bed height for video laryngoscopy coincided with that for mask ventilation. Video laryngoscopy offers enhanced flexibility in optimal patient positioning compared to Macintosh laryngoscopy, contributing to its advantages in tracheal intubation procedures.

## 1. Introduction

Airway management techniques, including mask ventilation and tracheal intubation, are essential not only in operating rooms, but also in diverse settings such as emergency rooms, general wards, and disaster sites. In other words, there are various places where patients may require airway management techniques. Therefore, these conditions may not always be optimal for airway management operators. These techniques are crucial because airway management failure is a major cause of cardiac arrest and anesthesia-related fatalities [1,2]. Airway management operators must be aware that the performance of their techniques is affected by the environment. Notably, during respiratory disease pandemics, anesthesiologists may need to perform these procedures outside the operating room [3,4].

In the operating room, airway management is a routine technique to ensure patient safety post-anesthesia induction. The height of the operating table significantly influences the performance of the airway management technique [5,6,7,8]. Therefore, it’s imperative to adjust the height of the operating table for each anesthesia procedure to optimize outcomes and prevent musculoskeletal disorders in operators [9,10,11,12]. While previous studies have explored the impact of operating table height on airway management techniques using Macintosh laryngoscopy, the influence on video laryngoscopy remains unexplored. Recently, various types of video laryngoscopes have become available, and the use of these devices must be discussed.

This study investigated the optimal operating table height for mask ventilation, laryngoscopic exposure, and tracheal intubation. We also compared the optimal operating table height for laryngoscopic exposure and tracheal intubation in direct and video laryngoscopy.

## 2. Materials and Methods

This study received approval from the Ethics Committee of Hiroshima University Hospital (approval number: E-2417). Anesthesiologists or specialists with significant airway management experience from our hospital participated in this study. Airway management techniques (mask ventilation, laryngoscopic exposure, and tracheal intubation) were conducted using a Laerdal Airway Management Trainer (Laerdal Medical Japan, Tokyo, Japan). A Macintosh laryngoscope (ACOMA Medical Industry, Tokyo, Japan) was utilized as a direct laryngoscope, while a McGRATH MAC (Aircraft Medical Ltd., Edinburgh, UK) and a Pentax Airway Scope (AWS-S200NK; Pentax Corporation, Tokyo, Japan) served as video laryngoscopes.

**Figure 1:**
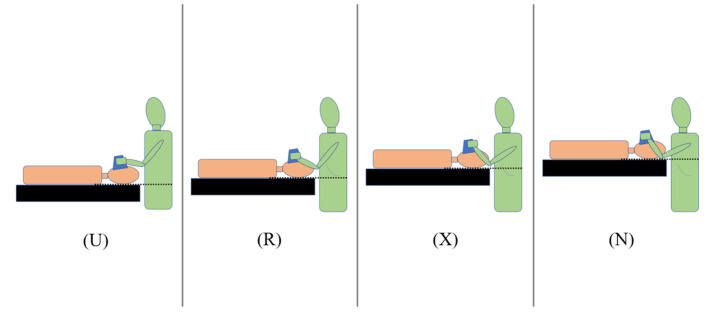
The height of the operating table. The height of the operating table was adjusted at the operator’s umbilicus (height U), inferior margin of the 12th rib (height R), xiphoid process (height X), and nipple (height N).

**Figure 2:**
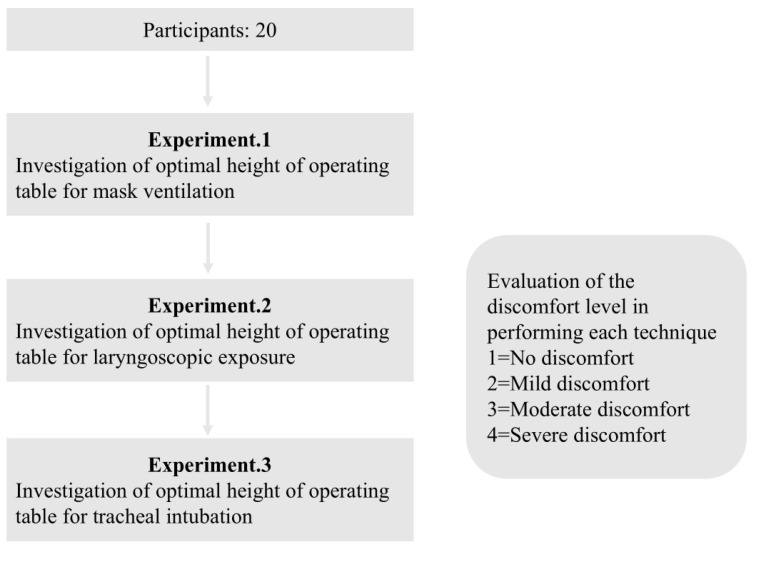
Study protocol and definition of “discomfort level”.

The height of the operating table was adjusted to the operator’s umbilicus (height U), inferior margin of the 12th rib (height R), xiphoid process (height X), and nipple (height N) (Figure 1). The techniques were performed with a “natural upright posture” without flexion of the neck, hips, or knees. In other words, the operator did not squat or bend during the procedure. The difficulty of each technique was subjectively rated by the operator on a 4-grade continuous “discomfort level”. (Evaluation criteria for each grade: grade 1 = no discomfort, grade 2 = mild discomfort, grade 3 = moderate discomfort, and grade 4 = severe discomfort) (Figure 2). We compared the total and the distribution of discomfort levels for each table height and device. The study comprised three parts: experiment (1), experiment (2), and experiment (3) (Figure 2).

### 2.1. Experiment (1)

The study explored the difficulty of mask ventilation at varying operating table heights (height U to N). The discomfort level during the procedure was evaluated at each table height.

### 2.2. Experiment (2)

The study examined the difficulty of laryngoscopic exposure using Macintosh, McGRATH, and AWS at different operating table heights (U–N). The discomfort level during the procedure was evaluated at each table height.

### 2.3. Experiment (3)

The study investigated the difficulty of tracheal intubation using Macintosh, McGRATH, and AWS at different operating table heights (U–N). The discomfort level during the procedure was evaluated at each table height.

### 2.4. Statistical Analysis

We compared the discomfort levels at each operating table height in two ways. First, the distribution of the difficulty of each technique was compared at the different heights (umbilicus, inferior margin of the 12th rib, xiphoid process, and nipple). Then, we aggregated the discomfort level scores at each table height and compared the aggregated scores at each height. A higher total discomfort level score at that table height indicated greater discomfort with that procedure. Statistical analysis employed Kruskal-Wallis and Steel-Dwass tests, with a significant set at *p* < 0.05.

## 3. Results

Twenty anesthesiologists or specialists with significant airway management experience participated in this study (Figure 2).

### 3.1. Experiment (1)

**Figure 3:**
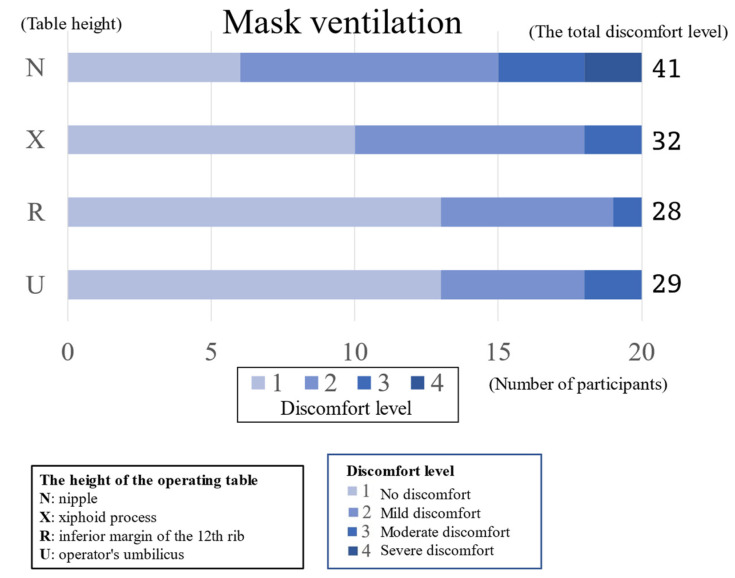
Difficulties in mask ventilation at different operating table heights. The figure shows the distribution of mask ventilation difficulty at each table height. The bar shows the distribution of discomfort levels for each height of the surgical table. Regarding the “discomfort level”, a higher- level value indicates that the procedure is more difficult. The number on the right side of the bar is the total score discomfort level of 20 anesthesiologists. Many anesthesiologists felt that mask ventilation at nipple level was more difficult than mask ventilation at any other height. The higher the operating table, the more anesthetists felt uncomfortable.

The results are shown in Table 1 and Figure 3. The total scores of the discomfort levels for mask ventilation were 41, 32, 28, and 29, for heights N, X, R, and U, respectively. There was a significant difference in the difficulty of mask ventilation with the height of the operating table; comparisons between the two groups showed significant differences between the umbilicus and the xiphoid process, the umbilicus and the nipple, the inferior margin of the 12th rib and the nipple, and the xiphoid process and the nipple.

The numbers show the distributions of the numbers of people by the level of discomfort during mask ventilation, laryngoscopic exposure, and tracheal intubation at each operating table height. The heights are represented by U: operator’s umbilicus, R: inferior margin of the 12th rib, X: xiphoid process, and N: nipple. The number of people for each level of discomfort was arranged in the following order: No discomfort, Mild discomfort, Moderate discomfort, Severe discomfort. Statistical analysis employed Kruskal-Wallis and Steel-Dwass tests, with a significant set at *p* < 0.05. Mask ventilation was perceived as easier at lower table heights. Conversely, direct laryngoscopic exposure was perceived as easier at higher table heights. The McGRATH laryngoscope showed consistent difficulty across table height, whereas the AWS tended to be somewhat more difficult at greater heights.

### 3.2. Experiment(2)

**Figure 4:**
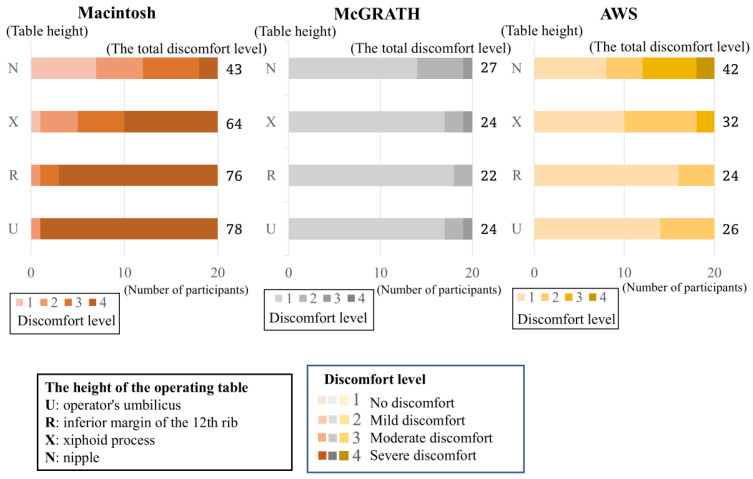
Difficulties in laryngoscopic exposure at different operating table heights. The figure shows the distribution of laryngoscopic exposure difficulty at each table height. The bars show the distribution of the discomfort levels for each height of the surgical table. Regarding the “discomfort level,” a higher-level value indicates that the procedure is more difficult. The numbers on the right side of the bars are the total scores for the discomfort levels of the 20 anesthesiologists. For the Macintosh laryngoscope, many anesthesiologists found the low height of the operating table difficult. For the video laryngoscope, many anesthesiologists found no difference in difficulty with the height of the operating table.

The results are shown in Table 1 and Figure 4. The total scores of the discomfort levels for laryngoscopic exposure with direct laryngoscopy were 43, 64, 76, and 78 for each height, respectively. The total scores of the discomfort levels for laryngoscopic exposure with McGRATH were 27, 24, 22, and 24 for each height, respectively. The total scores of the discomfort levels for laryngoscopic exposure with AWS were 42, 32, 24, and 26 for each height, respectively. For direct laryngoscopy, significant differences were observed in laryngoscopic exposure difficulty across operating table heights. Notably, comparisons between the groups showed significant differences between the umbilicus and xiphoid process, the umbilicus and nipple, the inferior margin of the 12th rib and nipple, and the xiphoid process and nipple. Conversely, the McGRATH study found no significant difference in laryngoscopic exposure difficulty at any operating table height. However, with the AWS, significant differences were noted in laryngoscopic exposure difficulty, particularly between the inferior margin of the 12th rib and the nipple.

### 3.3. Experiment (3)

**Figure 5:**
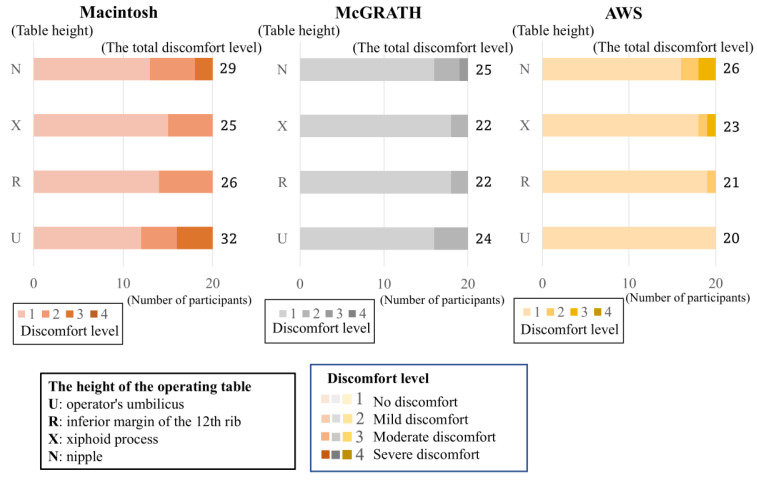
Difficulties in tracheal intubation at different operating table heights. The figure shows the distribution of tracheal intubation difficulty at each table height. The bars show the distribution of the discomfort levels for each height of the surgical table. Regarding the “discomfort level,” a higher-level value indicates that the procedure is more difficult. The numbers on the right side of the bars are the total score for the discomfort levels of the 20 anesthesiologists. Many anesthesiologists found no difference in intubation difficulty with the height of the operating table.

The results are shown in Table 1 and Figure 5. The total scores of the discomfort levels for tracheal intubation with direct laryngoscopy were 29, 25, 26, and 32 for each height, respectively. The total scores of the discomfort levels for tracheal intubation with McGRATH were 25, 22, 22, and 24 for each height, respectively. The total scores of the discomfort levels for tracheal intubation with AWS were 26, 23, 21, and 20 for each height, respectively. There was no significant difference in the difficulty of tracheal intubation in the free posture group. In the McGRATH study, there was no significant difference in the difficulty of tracheal intubation according to the height of the operating table. In the AWS, no significant difference was observed in the difficulty of tracheal intubation according to the height of the operating table. Given the challenge of intubation with the Macintosh laryngoscope in the upright position at low table heights, tracheal intubation difficulty was evaluated in the “free posture”.

## 4. Discussion

This study aimed to investigate the optimal height of the operating table that facilitates mask ventilation, tracheal intubation using a direct laryngoscope, and tracheal intubation with a video laryngoscope. This study found that mask ventilation was more difficult at higher operating table heights. The total scores of discomfort levels for mask ventilation were lowest when the operating table height was the inferior margin of the 12th rib. In contrast, laryngoscopic exposure with direct laryngoscope was easier at higher operating table heights. The total scores of discomfort levels for laryngoscopic exposure with direct laryngoscope were lowest when the operating table height was nipple. Laryngoscopic exposure with the McGRATH showed consistent ease across table heights, while with the AWS, it tended to be more difficult at a greater height. No variation in tracheal intubation difficulty using a video laryngoscope was observed between the McGRATH and the AWS.

Tracheal intubation typically involves a series of airway management techniques, each requiring an appropriate operating table height. However, the advantages and disadvantages of adjusting this height have not yet been fully discussed. In our study, the height of the operating table suitable for laryngoscopic exposure using the Macintosh laryngoscope was higher than the height of the operating table suitable for mask ventilation. Therefore, when performing tracheal intubation using a direct laryngoscope, it was better to elevate the operating table after mask ventilation to avoid straining the posture and to achieve the best performance. However, the optimal operating table height for video laryngoscopes (McGRATH and AWS) was similar to that for mask ventilation, revealing that there was no need to adjust the operating table when using these devices for laryngoscopic exposure.

Video laryngoscopy has been reported to be useful for tracheal intubation of difficult airways [13,14,15]. Our study showed that video laryngoscopy facilitated lower laryngoscopic exposure compared to direct laryngoscopy, suggesting its utility in settings where bed height cannot be adjusted, such as general wards and emergency rooms [16,17,18,19]. COVID-19, which has become a global pandemic, has been reported to cause rapid deterioration of respiratory status, and it is expected that there will be more opportunities for tracheal intubation in general hospital wards [3,4]. If the intubator is not accustomed to intubating the trachea outside the operating room, it is important to understand the characteristics of each laryngoscope and choose a video laryngoscope.

The height of the operating table affects the performance of anesthesia techniques [5,6,7,8]. Therefore, the height of the operating table must be adjusted for each anesthesia technique. Additionally, anesthesia techniques performed in awkward postures can cause musculoskeletal disorders [9,10,11]. Anesthesiologists have been reported to have more musculoskeletal disorders than other professions [10,12].

During anesthesia induction, anesthesiologists handle various tasks, including drug administration, airway management, circulation management, and monitoring, with tracheal intubation being particularly stressful [20]. Fatigue caused by stress loads during anesthesia induction can affect subsequent anesthesia management. Therefore, it is necessary to reduce stress during the induction of anesthesia.

The McGRATH and AWS video laryngoscopes were used in this study. They are the most widely used video laryngoscopes in Japan. In the McGRATH study, no significant difference was observed in the difficulty of laryngoscopic exposure and intubation according to the height of the operating table. In the AWS group, laryngoscopic exposure was slightly difficult when the operating table was higher. One possible reason for this is the difference in blade geometry between McGRATH and AWS. The AWS blade exhibits a near-right-angle bend. Therefore, the blade must be inserted further in front of the patient when inserted into the oral cavity. When the operating table is high, the operator’s shoulder joint must be rotated outward, and the operator’s elbow must be raised to insert the blade. Many anesthesiologists find this uncomfortable.

This study has some limitations. First, we used a tracheal manikin to estimate patient-induced intubation difficulties. In other words, some of the research data obtained from manikin models may not be directly applicable to clinical patients because the difficulty of laryngeal deployment in manikin models does not perfectly match that in clinical patients. In contrast, we are currently investigating the posture during mask ventilation and tracheal intubation. Therefore, we believe it is more appropriate to use a manikin model with fewer individual differences for tracheal intubation. Second, we used ”discomfort level” to assess the difficulty of the airway management techniques. “Discomfort level” indicates stress on the airway management technique and is not necessarily indicative of the difficulty of the airway management technique.

## 5. Conclusions

This study utilized a tracheal intubation manikin and mask ventilation was easiest to perform when the operating table height was at the inferior margin of the 12th rib. Furthermore, laryngoscopic exposure using a direct laryngoscope was facilitated at the level of the nipple. With video laryngoscopy, there was no difference in the difficulty of tracheal intubation depending on the operating table height, and laryngoscopic exposure did not pose challenges, even at lower table positions. Thus, while direct laryngoscopy necessitates posture adjustments for optimal tracheal intubation following mask ventilation, video laryngoscopy does not require such alterations. We believe that these unique features of laryngoscopes are important factors in choosing a laryngoscope in situations where the patient’s height cannot be adjusted, such as emergency departments and general wards.

## Figures and Tables

**Table 1:** Distribution of discomfort levels of mask ventilation, laryngoscopic exposure, and tracheal intubation at each operating table height. Significant differences (*p* < 0.05) are written in red.

	Operating Table Height				*p* Value						
Mask Ventilation	U	R	X	N	Overall	U vs. R	U vs. X	U vs. N	R vs. X	R vs. N	X vs. N
	13/5/2/0	13/6/1/0	10/8/2/0	6/9/3/2	<0.001	0.062	0.002	<0.001	0.351	<0.001	0.006
	**Operating Table Height**				***p* Value**						
**Laryngoscopic Exposure**	**U**	**R**	**X**	**N**	**Overall**	**U vs. R**	**U vs. X**	**U vs. N**	**R vs. X**	**R vs. N**	**X vs. N**
Macintosh	0/1/0/19	0/1/2/17	1/4/5/10	7/5/6/2	<0.001	0.757	0.013	<0.001	0.080	<0.001	0.015
McGRATH	17/2/1/0	18/2/0/0	17/2/1/0	14/5/1/0	0.390	0.955	1.000	0.714	0.955	0.381	0.714
AWS	14/6/0/0	16/4/0/0	10/8/2/0	8/4/6/2	0.007	0.889	0.471	0.057	0.163	0.017	0.470
	**Operating Table Height**				***p* Value**						
**Tracheal Intubation**	**U**	**R**	**X**	**N**	**Overall**	**U vs. R**	**U vs. X**	**U vs. N**	**R vs. X**	**R vs. N**	**X vs. N**
Macintosh	12/4/4/0	14/6/0/0	15/5/0/0	13/5/2/0	0.549	0.733	0.555	0.958	0.985	0.952	0.833
McGRATH	16/4/0/0	18/2/0/0	18/2/0/0	16/3/1/0	0.459	0.818	0.818	0.970	1.000	0.582	0.582
AWS	20/0/0/0	19/1/0/0	18/1/1/0	16/2/2/0	0.138	0.749	0.479	0.160	0.925	0.461	0.820

## Data Availability

The data presented in this study are available on request due to legal reasons.
